# Prevalence and Farm Management Factors and Milk Quality Changes Related to Bovine Mastitis in Small‐ and Medium‐Sized Farms in the Central Highlands of Ecuador

**DOI:** 10.1155/vmi/6213804

**Published:** 2025-12-29

**Authors:** Alicia Maya-Delgado, Lenin Ron-Garrido, Katherine Balarezo-Espinoza, María J. Poveda-Tutasi, Cristina Cholota-Iza, María A. Chávez-Larrea, Armando Reyna-Bello, Sarah Martin-Solano, Claude Saegerman, Jorge Ron-Román

**Affiliations:** ^1^ Department of Life Sciences and Agriculture, Army University of Ecuador (ESPE), Sangolquí, 171-5-231, Ecuador; ^2^ Fundamental and Applied Research for Animal and Health Center, Faculty of Veterinary Medicine, University of Liège, Liège, 4000, Belgium, ulg.ac.be; ^3^ Institute for Zoonosis Research (CIZ), Central University of Ecuador, Quito, 170521, Ecuador, uce.edu.ec; ^4^ Faculty of Veterinary Medicine and Zootechnics, Central University of Ecuador, Quito, 170521, Ecuador, uce.edu.ec

**Keywords:** bovine mastitis, dairy cow, Ecuador, management, milking practices, protective factors, risk factors

## Abstract

The Ecuadorian highlands provide favourable climate conditions for bovine milk production, where small‐ and medium‐sized farms represent over 80% of dairy producers. However, in communities with limited technological resources, both clinical (CM) and subclinical mastitis (SM) pose significant challenges to productivity. This study surveyed a dairy‐producing community in the central Ecuadorian highlands to evaluate risk factors associated with bovine mastitis and to analyse milk quality parameters. The prevalence of SM and CM at the animal level was 34.8% (130/374) and 1.9% (7/374), respectively, and at the farm level, it was 34.1% (29/85) for SM and 7.0% (6/85) for CM. Univariate and multivariable logistic regression models were used to identify factors associated with the presence of SM and CM. An associated factor with the presence of SM (*p* < 0.05) was the non‐compliance with time medication withdrawal (odds ratio [OR] = 3.88; 95% CI: 1.31–13.0). In contrast, performing the California mastitis test was a protective factor (OR = 0.32; 95% CI: 0.10–0.93). At animal level, the use of crossbreeds increased the probability of SM (OR = 2.64; 95% CI: 1.58–4.41). The presence of SM in the herd significantly increased the risk of CM (OR = 12.47; 95% CI: 1.30–119.31). Milk samples positive for mastitis showed changes in density, added water, and freezing point. These findings highlight the need for farmer training and strict adherence to diagnostic and preventive protocols for bovine mastitis in order to improve herd health and milk quality.

## 1. Introduction

Mastitis, the inflammation and infection of the mammary gland, is a multifactorial disease affecting cows in the dairy industry. Mastitis has a worldwide distribution, affecting dairy industry in both developed and developing countries [1, 2]. Mastitis draws particular attention from animal health professionals worldwide due to the observed changes in milk composition, reduction of milk quality and quantity, and because of economic losses related to low production and cost of the treatments [3–6]. Mastitis is traditionally classified as subclinical (SM) or clinical (CM), depending mainly on the presence and severity of clinical signs. In herds with a high prevalence of CM, poor‐quality milk is often discarded, and persistently affected cows may be culled, generating further economic losses for farmers [7].

In South America, estimated losses due to bovine mastitis were about USD 0.06 per dairy cow per day in Argentina and USD 0.19 per dairy cow per day in Colombia [8, 9]. In Ethiopia, the overall financial loss per lactation was estimated at USD 78.65 [10], while in Nigeria, the losses range from USD 0.23 and USD 0.42 per dairy cow/day [11]. In United States of America, losses were estimated at approximately USD 0.44 to USD 0.94 per dairy cow/day [10]. In Ecuador, the economic impact caused by mastitis has not yet been quantified.

In Ecuador, there are approximately 249,000 milk producers; most of them (80%) are smallholder farmers. The national milk production is around 6.65 million litres per day, generated by 996,503 dairy cows with an average yield of 5.6 L/cow/day [12]. Assuming cows are lactating 80% of the year, the average yield increases to 8.34 L/cow/day. Dairy industries in Ecuador are located primarily in the highlands of Ecuador, mainly because of the presence of dairy cattle breeds. According to the National Office of Statistics (INEC), of the total milk produced daily in the country, 11.6% is destined for pasteurisation and consumption as fresh milk; 6.2% is used for the production of pasteurised dairy products; 27% is consumed in the form of raw milk, either directly or in artisanal products; and 31.7% is destined for self‐consumption on farms, mostly in the form of milk to feed calves [12]. In terms of commercial trade in 2021, on the one hand, Ecuador imported dairy products for the sum of USD 29,177,577, mainly due to the import of infant formula. On the other hand, between 2019 and 2021, the country exported USD 3,390,538 in products, with milk powder, cheese and liquid milk being those products mainly destined to Peru, Colombia, Venezuela, Bolivia and United States [12, 13].

In Ecuador, local studies have reported a prevalence of bovine mastitis ranging from approximately 60% to 64% at the animal level [14, 15]. However, there are not scientific studies that could determine or evaluate the potential risk or protective factors associated with the persistence of mastitis in cattle. Proper sanitary management and continuous monitoring on dairy farms, especially in vulnerable sectors, can lead to positive changes in milk production. Moreover, understanding the epidemiology of disease is essential for identifying and understanding the factors associated with its occurrence, and it is also a necessary condition for the prevention and control of bovine mastitis [16, 17]. Housing, hygiene, seasonal conditions and management during both milking and dry periods are important aspects to consider for maintaining udder health and ensuring milk quality and production [6].

In this study, we established three objectives: (1) to determine the prevalence of clinical (CM) and subclinical mastitis (SM) in cattle; (2) to assess milk quality through physical–chemical parameters; and (3) to identify potential risk factors associated with bovine mastitis in the central part of the Ecuadorian highlands. In addition, we investigated a possible relationship between anaemia, hypoproteinaemia and mastitis. The epidemiological insights generated by this study, conducted in a context where smallholder dairy production predominates, illustrate the mastitis problem in the area and will support the development of prevention, control and training strategies adapted to local realities.

## 2. Materials and Methods

### 2.1. Study Area and Sampling Design

The study was conducted between 2018 and 2020 in a dairy farming community located in the central highlands of Ecuador, with a population of approximately 8000 people. The community’s economy is based on the production of milk and dairy products such as fresh and mature cheese and butter, which are sold locally, nationally and internationally. To ensure that the results of the study do not negatively affect the community, the exact location and name of the community were kept confidential. The area spans two climatic zones: páramo and subtropical, ranging from 800 to 4800 m above sea level. The average annual temperature is 13.5°C, the average annual precipitation is 845 mm, and the relative humidity is 90% [18].

Due to the incomplete sampling frame of farmers, a snowball sampling strategy was employed [19]. Through socialisation meetings, authorities and community leaders facilitated the recruitment of farmers, with an emphasis on highlighting small‐ and medium‐sized farms. Of the 120 dairy farms in the survey area, 85 farms (71%) participated in the study. Farms were classified according to the number of cattle present at the time of the visit: small (1–20 cattle; 76 farms) and medium (21–70 cattle; 9 farms). Milk and blood samples were collected from all milking cows on each farm prior to milking. In total, 374 cows were sampled, with an average age of 63.4 months (standard error: 27.6 months) and a median age of 60 months.

### 2.2. Identification of Bovine Mastitis

Milk samples for quality determination were collected during milking. In the absence of cattle chutes or other restraining facilities, cows were restrained by tying a rope around the hind legs. Teats were cleaned with water and dried with a paper towel prior to sampling.

Two field procedures were used for the diagnosis of bovine mastitis: (1) the black strip cup (BSC) test to detect CM and (2) the California mastitis test (CMT) to detect SM. Both field tests were performed according to the procedures described by the Canadian Bovine Mastitis Research Network [20], following the standardised interpretation criteria [21, 22].

For the diagnosis of CM, the BSC test was used. This method detects physical changes in milk such as discoloration, and the presence of clots, blood or pus. The first three streams of milk from each udder quarter were collected in a dark cup and examined for these changes. SM was diagnosed using the CMT. For each quarter, 3 mL of milk was placed in a well of the test plate and mixed with 3 mL of CMT reagent. The plate was rotated for approximately 10 s, and the reaction was assessed. The CMT result is based on the increased leukocyte concentration, which leads to gel formation, and is predictive of the somatic cell count.

### 2.3. Physicochemical Quality of Milk

To assess milk quality, 50 mL of composite samples (pooled from all four‐quarters) were collected in sterile bottles with tight‐fitting lids. Cows were identified using field records. Samples were stored in insulated boxes with ice packs at 4°C and transported to the laboratory.

We assessed milk quality in a laboratory temporarily installed on the premises of the main cheese factory, located in the rural community of the study area, using the following parameters: fat, protein, solids not fat, lactose, density, freezing point, and added water in fresh milk, using the Ekomilk Bond equipment [23].

A microprocessor in the Ekomilk equipment translates the results by measuring the explained parameters taking as a reference the density, which is a variable that determines the relationship between the mass and the volume of a substance. The density of milk is therefore directly related to the amount of fat, non‐fat solids and the amount of water added to the milk [23].

### 2.4. Haematological and Biochemical Blood Tests

Blood samples were collected from the caudal vein of each animal into tubes with and without anti‐coagulant (EDTA). Haematocrit was determined using capillary tubes centrifuged at 20,000 rpm for 5 min, and values were read with a micro‐haematocrit reader. Serum was separated by centrifugation (3500 rpm for 5 min), transferred to plastic tubes and stored for further analyses. The total protein concentration (g/mL) in serum was measured using a refractometer.

### 2.5. Data Collection and Statistical Analysis

Two tools were used to collect information: (1) an epidemiological survey administered to each farm in the study and (2) a sampling record for each animal sampled. The epidemiological survey included the following sections: (1) farm identification and location, (2) general farm data (land tenure system, area, animal inventory), (3) health management (biosecurity, drinking water and feed management, manure management, origin of replacement animals and quarantine, deworming and vaccination schedule, veterinary care), (4) reproductive management (breeding system, existence of pathologies), (5) production management (milking system, diagnosis and treatment of mastitis) and (6) knowledge of bovine mastitis.

The animal sampling record included (1) animal and sample identification; (2) zootechnical data (sex, age, breed); (3) clinical aspects of mastitis (body temperature, pain in the mammary gland, teat hyperkeratosis); and (4) results of the BST and CMT field tests. Additional records were used to document laboratory results on parameters related for milk quality parameters. To ensure the accuracy and data integrity, field and laboratory results were exported to the Microsoft Excel sheet for the purpose of verification, organisation and data cleaning.

The CMT and BST results were categorised on a numerical scale ranging from 0 to 4 to detect the presence of mastitis and distinguish between CM and SM. A score of zero means no presence of mastitis, a score of 4 means the presence of CM and with a score of 1–3 means the presence of SM [21, 24]. For the dependent variable, in the case of SM, a farm was declared positive when the average of the CMT scores of the sampled animals was higher than 1; only the scores of the animals ranging from 1 to 3 were taken into account to average this value. In the case of CM, the presence of at least one cow with a CMT score of 4 and positive to BST was sufficient to declare farms as positive.

The potential factors analysed were related to milking management practices on the farm and included durability of the milking equipment; adherence to the recommended sequence of the milking protocol; availability of water for regular cleaning of equipment and udders; frequency of veterinary visits; and compliance with the withdrawal period for milk excluded from commercial sale due to antibiotic treatment. Most factors were recorded as binary variables (yes = present/compliant; no = absent/noncompliant). Veterinary care was categorised as high (at least once per month), low (less than once per month) or none.

Before model fitting, covariates were screened for variability. Variables with low variability (e.g., > 95% of observations sharing the same value) were excluded from further analysis due to a lack of discriminatory power. For the comparison of milking practices at the farm level and the presence of the CM and SM in the study area, we used a univariate logistic regression. Then, at the farm level, a multivariable logistic regression model was used to combine and evaluate covariates. We used the odds ratio (OR) as a measure of association between compliance or not of the factor and the outcome of the presence of mastitis. Thus, the OR is interpreted as an exploratory variable indicating the probability of what happens with the presence or absence of mastitis [25, 26]. The variance inflation factor (VIF) was used to determine the degree of multicollinearity between the covariates. The VIF function of the REGCLASS package in R was used [27]. When the VIF value was greater than 5, it suggests the existence of multicollinearity [28]. To identify the covariates in the final model, we performed a stepwise procedure with a multivariable logistic regression using the step.AIC function from the MASS package in the R environment [28], and variables with a *p* value of less than 0.30 in the univariate analysis were kept for the final model. An additional variable CMT (specific for SM) was also retained (*p* value = 0.33).

For the analysis of explanatory factors at the animal level, we included two variables (age, breed of dairy cows). The glmer function of the lme4 package in the R environment [27] was used to incorporate both fixed‐effects parameters and random effects (farm to which they belong) into a linear predictor, using maximum likelihood. The risk or protective explanatory variables with a *p* value ≤ 0.05 were associated with the presence of SM and CM. We performed a comparison between milk samples for cows with and without SM in relation to milk quality characteristics and health parameters, such as animal temperature and blood tests including haematocrit and total blood serum protein through a mixed model in which the SM status of cows was the fixed effect and the herd was the random effect.

All risk and protective explanatory variables were weighted by their OR into a single overall weighted score (OWS) at the farm level for the SM. The area under the receiver operating characteristic curve (AUC‐ROC) was used to measure performance for the OWS classification. We obtained the Youden index and ROC curve analysis using the R environment and the web tool: Easy ROC; used to estimate the best cut‐off point. The Youden index was calculated to evaluate the performance of OWS on farms with the presence and without the presence of SM. This Youden index was defined as sensitivity + specificity − 1 [29]. The identification of inadequate practices in the prevention, diagnosis and treatment of mastitis, as well as the identification and assessment of risk and protective factors related to bovine mastitis, allowed guiding and preparing a training strategy for the producers.

### 2.6. Ethical Aspects

Due to the nature of the study and the low risk to participants, no formal Ethics Committee approval was required. All animals were treated with care, and the usual farm management of sample collection was followed, without mistreatment and ensuring animal welfare. The project was also validated and supported by the University ESPE, the NGO Via Don Bosco and the cooperative of producers from the centre highlands of Ecuador, namely, PRODUCOOP.

The farmers were properly informed and gave their written informed consent prior to sampling their animals.

## 3. Results

Women represented 45.8% (39/85) of farm owners and 68.2% (58/85) of those responsible for managing dairy herds. These figures highlight the central role of women in herd management and decision‐making within the study area, underscoring the importance of integrating a gender perspective into livestock development strategies.

### 3.1. Prevalence of Bovine Mastitis

The herd prevalence of SM was 34.1% (95% CI: 23.9–44.7), and the herd prevalence of CM was 7.0% (95% CI: 2.6–14.6). At the animal level, using CMT, the SM prevalence was 34.8% (95% CI: 29.9–39.8), and using the black strip cup test (BST) and CMT, the CM prevalence was 1.9% (95% CI: 0.8–3.8). Table 1 shows the prevalence and the number of farms and animals reported with SM and CM in the central highlands of Ecuador.

**Table 1:** Prevalence of bovine mastitis at farm and animal levels.

	No mastitis positive	Clinical mastitis	Subclinical mastitis	Total
Farm level	58.8% (50/85)	7.0% (6^∗^/85)	34.1% (29/85)	85
Cow level	63.4% (237/374)	1.9% (7/374)	34.8% (130/374)	374

^∗^We found both subclinical and clinical mastitis on six farms.

### 3.2. Identification of the Potential Risk Factors of SM in Herds

In a first step, we performed a univariate analysis with 15 variables (Table 2). We identified two risk factors for SM: the non‐compliance with drug withdrawal time (OR [OD] = 3.03 with 95% CI: 1.45–7.98) and the presence of CM in the herd (OR = 12.47 with 95% CI: 1.30–119.31). In addition, the frequency of veterinary assistance in the farm was protective factor (OR = 0.27 with 95% CI: 0.07–0.99).

**Table 2:** Milking management characteristics potentially associated with subclinical mastitis at the farm level using a univariate analysis.

Explanatory variable	Modality	Number of farms	Positive farms (%)	OR (95% CI)	*p* value
Resistant utensils for the milking	No	46	19 (41.3)	Reference	
	Yes	39	10 (25.6)	0.49 (0.19–1.24)	0.13

Sequence of milking protocol	No	32	8 (25.0)	Reference	
	Yes	53	21 (39.6)	1.97 (0.75–5.20)	0.17

Pre‐milking teat washing	No	11	5 (45.5)	Reference	
	Yes	74	24 (32.4)	0.58 (0.16–2.08)	0.40

Enough water for proper milking procedures	No	60	18 (30.0)	Reference	
	Yes	25	11 (44.0)	1.83 (0.70–4.81)	0.22

Discard the first milk jet	No	28	11 (39.3)	Reference	
	Yes	57	18 (31.6)	0.71 (0.28–1.83)	0.62

Teat dipping	No	80	26 (32.5)	Reference	
	Yes	5	3 (60.0)	3.07 (0.33–38.86)	0.48

Frequency of veterinary assistant	High	15	8 (53.3)	Reference	
	Low	30	7 (23.3)	0.27 (0.07–0.99)	0.05^∗∗^
	Null	40	14 (35.0)	0.47 (0.14–1.57)	0.22

Separating animals affected with mastitis	No	35	8 (22.9)	Reference	
	Yes	50	21 (42.0)	2.44 (0.93–6.44)	0.07

Performing CMT	No	35	14 (40.0)	Reference	
	Yes	50	15 (30.0)	0.64 (0.26–1.59)	0.34

Farm size	Medium	9	3 (33.3)	Reference	
	Small	76	26 (34.2)	1.04 (0.24–4.50)	0.96

Manure presence during milking	No	12	3 (25.0)	Reference	
	Yes	73	26 (35.6)	1.66 (0.41–6.67)	0.48

Mechanised milking	No	82	27 (32.9)	Reference	
	Yes	3	2 (66.7)	4.07 (0.35–46.94)	0.26

Training in milking	No	42	12 (28.6)	Reference	
	Yes	43	17 (39.5)	1.63 (0.66–4.05)	0.29

Non‐compliance with medication withdrawal time	No	38	8 (21.1)	Reference	
	Yes	47	21 (44.7)	3.03 (1.45–7.98)	0.025^∗^

Clinical mastitis	Neg	80	25 (30.9)	Reference	
	Pos	5	4 (80.0)	12.47 (1.30–119.31)	0.029^∗^

*Note:* %: percentage; Neg: negative; Pos: Positive.

Abbreviations: CI, confidence interval; OR, odds ratio.

^∗^Risk factor.

^∗∗^Protective factor.

For the multivariate model, we initiated a logistic regression model with 10 out of 15 variables. Variables with a *p* value less than 0.30 and an additional variable CMT (specific for SM) were chosen, and they were resistant utensils for milking, sequence of milking protocol, frequency of veterinary assistance, performing CMT, not compliance with medication withdrawal time, enough water for proper milking procedures and separating animals affected with mastitis. Collinearity was minimal in the set of variables, with all GVIF‐adjusted values remaining well below concerning levels, indicating that the predictors function independently within the epidemiological model (GVIF factors presented in Supporting information Table S1). Table 3 indicates the final model and the number of animals contained in each category with the respective positive animals. The first model presented an Akaike information criterion [AIC] = 112. The final model (AIC = 105.7) contained three significant variables (Table 3).

**Table 3:** Associated factors found in the final multivariable logistic regression model for subclinical mastitis at the farm level analysis.

Characteristic	OR (95% CI)	*p* value
Resistant utensils for the milking^a^	Reference	
	0.43 (0.14–1.29)	0.13

Sequence of milking protocol^b^	Reference	
	2.57 (0.85–7.77)	0.10

Frequency of veterinary assistance^c^	Reference	
	0.21 (0.05–0.90)	0.036^∗∗^
	0.41 (0.10–1.61)	0.20

Performing CMT^d^	Reference	
	0.32 (0.11–0.96)	0.042^∗∗^

Non‐compliance with medication withdrawal time^e^	Reference	
	3.88 (1.25–12.1)	0.019^∗^

*Note:* %: percentage.

Abbreviations: CI, confidence interval; OR, odds ratio.

^a^Aluminium or stainless steel (corrosion‐resistant).

^b^Correct sequence of the milking process.

^c^Assistance in general aspects such as vaccination, physical accidents of the animals, vitamin administration and not specific for bovine mastitis.

^d^California mastitis test regularly used and before the milking process.

^e^Deliver milk in the cheese factory or direct consumption of the milk without respecting the waiting time of the medication applied to the cow.

^∗^Risk factor.

^∗∗^Protective factor.

We identified two protective factors: the low frequency of veterinary assistance in the field (OR = 0.21 with 95% CI: 0.05–0.88) and the application of CMT before milking (OR = 0.32 with 95% CI: 0.10–0.93). On the other hand, the non‐compliance with medication withdrawal time (OR = 3.88 with 95% CI: 1.31–13.0) appeared as a risk factor. At the animal level, the age of the animal was not associated with SM; only the animal breed showed a significant association (*p* value < 0.05). Crossbreeds have a higher risk of SM (OR = 2.64, 95% CI: 1.58–4.41) compared to purebreds or exotic breeds.

With respect to non‐compliance with milk withdrawal periods, the study found that sanitary records for mastitis treatment were not used. Instead, producers relied on memory to recall which animals were treated, when treatment began and which drugs were administered. As a result, withdrawal periods were often disregarded, and milk from treated animals was not consistently excluded from sale. Furthermore, farmers did not separate infected animals, thereby maintaining active sources of infection within the herd.

### 3.3. Identification of Risk Factors of CM at Herd Level

The same procedure for the analysis of SM was applied for CM (Table 4). Just one risk factor was identified, the presence of SM in the herd (OR = 12.47 with 95% CI: 1.30–119.31). We found low significance in the other variables because there were insufficient observations and a few clinical cases. That is why the multivariable logistic regression model was not performed for CM.

**Table 4:** Characteristic of the milking management associated with clinical mastitis at the farm level using univariate analysis.

Explanatory variable	Modality	Number of farms	Positive farms (%)	OR (95% CI)	*p* value
Resistant utensils for the milking	No	50	1 (2.50)	Reference	
	Yes	35	4 (11.43)	5.05 (0.47–258.41)	0.17

Sequence of milking protocol	No	39	2 (6.90)	Reference	
	Yes	46	3 (6.52)	0.90 (0.09–11.36)	1

Pre‐milking teat washing	No	20	1 (10.00)	Reference	
	Yes	65	4 (6.15)	0.57 (0.04–30.92)	0.50

Enough water for proper milking procedures	No	62	2 (3.85)	Reference	
	Yes	23	3 (13.04)	3.88 (0.41–49.38)	0.15

Discard the first milk jet	No	35	2 (8.00)	Reference	
	Yes	50	3 (6.00)	0.73 (0.11–4.70)	0.74

Teat dipping	No	81	4 (5.63)	Reference	
	Yes	4	1 (25.00)	4.59 (0.07–65.84)	0.26

Frequency of veterinary assistance	High	24	2 (14.29)	Reference	
	Low	26	2 (7.69)	0.60 (0.03–7.82)	0.51
	Null	35	1 (2.86)	0.18 (0.02–3.83)	0.19

Separation of animals contaminated with mastitis	No	43	1 (3.03)	Reference	
	Yes	42	4 (9.52)	2.92 (0.27–149.70)	0.64

Performing CMT	No	41	3 (9.68)	Reference	
	Yes	44	2 (4.55)	0.44 (0.03–4.14)	0.39

Farm size	Medium	18	1 (12.50)	Reference	
	Small	67	4 (5.97)	0.45 (0.03–24.59)	0.43

Manure presence during milking	No	22	1 (8.33)	Reference	
	Yes	63	4 (6.35)	0.64 (0.05–7.32)	0.54

Mechanised milking	No	83	4 (5.48)	Reference	
	Yes	2	1 (50.00)	9.14 (0.13–214.25)	0.16

Training in milking	No	49	3 (7.69)	Reference	
	Yes	36	2 (5.56)	0.63 (0.05–5.87)	0.67

Non‐compliance with time medication withdrawal	No	46	1 (2.78)	Reference	
	Yes	39	4 (10.26)	3.39 (0.31–173.84)	0.37

Subclinical mastitis	Neg	64	1 (1.82)	Reference	
	Pos	21	4 (19.05)	12.47 (1.30–119.31)	0.029^∗^

*Note:* %: percentage; Pos: Positive; Neg: Negative.

Abbreviations: CI, confidence interval; OR, odds ratio.

^∗^Risk factor.

^∗^Significance of *p* value < 0.05.

### 3.4. Quality of the Milk

Milk quality characteristics and a basic haematocrit of the milking cows with and without SM are presented in Table 5. We observed significantly low values in the SM group for the percentage of protein, the percentage of solid non‐fat and the freezing point of milk. Significantly higher values were observed in SM cows for blood total serum protein, density, percentage of water in milk and the freezing point.

**Table 5:** Relationship between the characteristics of quality milk and the presence (*N* = 130) or absence (*N* = 244) of subclinical mastitis.

Characteristics (unit)	Presence of subclinical mastitis	Mean	SD	Difference	*t-*value	*p* value
Haematocrit (mL/mL#)	Neg	34.71	4.17	0.83	1.43	0.18
	Pos	35.5	4.38			

Blood total serum protein (g/L)	Neg	7.93	8.00	0.20	2.26	0.02^∗^
	Pos	8.13	8.20			

Temperature (°C)	Neg	37.51	0.80	0.07	1.67	0.10
	Pos	37.58	0.70			

Protein (%) in milk	Neg	3.21	0.24	0.15	0.55	0.59
	Pos	3.06	0.26			

Solids not fat (%)	Neg	8.45	0.70	0.02	1.29	0.19
	Pos	8.03	0.76			

Fat (%)	Neg	4.42	2.52	0.92	1.61	0.11
	Pos	5.34	2.84			

Density (g/cm^3^)	Neg	1.029	0.004	0.002	3.46	0.00^∗∗^
	Pos	1.027	0.006			

Water in milk (%)	Neg	3.46	4.43	2.46	3.09	0.00^∗∗^
	Pos	5.92	5.65			

Freezing point (°C)	Neg	−0.55	5.18	−0.03	3.80	0.00^∗∗^
	Pos	−0.52	6.24			

*Note:* Neg, negative; Pos, positive; l/l, litre of cells per litre of blood.

Abbreviation: SD, standard deviation.

^∗^Significance of *p* value < 0.05.

^∗∗^Significance of *p* value < 0.01.

### 3.5. OWS at the Farm Level Under the Receiver Operating Characteristic Curve (ROC)

We calculate the OWS at the farm level using all the variables included in the final model using the following formula:

(1) OWS=IFA=“High”;4.775;or IFA=“Null”;2.4520; +2.3262.5673.1253.884×IFB=01;+×C+×IFD=10;+×IFE=10;,

Where:

• A is 1 ∗ 4.775 if the veterinary assistant’s frequency was high, or 1 ∗ 2.45 if their frequency is null, or 0 otherwise.
• B is 1 ∗ 2.326 if the farmer uses resistant milking utensils, or 0 otherwise.
• C is 1 ∗ 2.567 if the farmer follows the milking protocol sequence, or 0 otherwise.
• D is 1 ∗ 3.125 if the farmer does not adhere to medication waiting times, otherwise 0.
• E is 1 ∗ 3.884 if the farmer performs CMT, otherwise 0.

**Figure 1:**
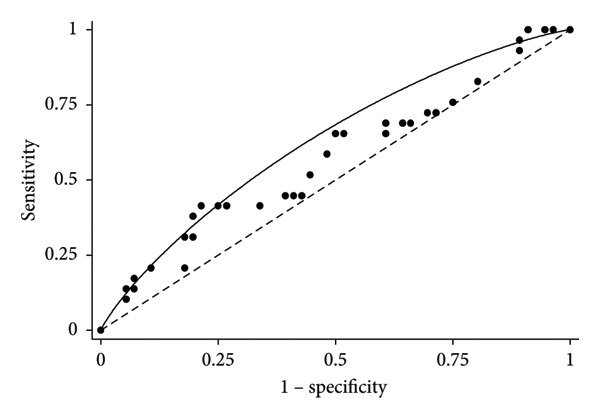
Receiver operating characteristic curve of the overall weighted score (OWS) of a high level of subclinical mastitis. The dot line indicates the limit of discrimination; when the OWS is below this line, the discriminatory power of the OWS is bad (not useful model). Points are the observed values; the solid curve in black was fitted according to a binormal distribution. Area under the curve = 0.63 (95% CI: 0.504–0.76) with standard error = 0.065.

The minimum and the maximum theoretical values of the OWS are 2.33 and 14.35, respectively. The probability that a farm has SM is in function of the OWS. We assessed the diagnostic discriminatory power of OWS by calculating the AUC–ROC after fitting it, and it was equal to 0.63 (95% CI: 0.504–0.76) with a standard error of 0.065 (Figure 1). Through the Youden index, the optimal cut‐off to discriminate the presence of SM was OWS = 9.58. Implementing this cut‐off, the model sensitivity was 41.4% and the model specificity was 78.6%.

## 4. Discussion

Dairy production is an important economic activity in the central highlands of Ecuador. In the 1970s, a solidarity economy model began promoting dairy production and prioritised the industrialisation of its products for not only livestock farming but also agriculture. However, such an economic activity requires support in training and empowerment [30, 31].

In this survey, women have a significant presence in dairy farming. The women who own the small‐ and medium‐sized farms are also the farm managers. According to FAO (2022), dairy practices, and specially the milking, in the majority of small dairy farmers in developing countries, are manual and they are an activity that is traditionally carried out by women [32].

The prevalence of SM is always higher than that of CM despite the differences in management, environmental conditions and technology provided by the farmer [33, 34]. Heider [35] estimates that for every case of CM observed, there are 15–40 cases of SM in the herd. CM is easier to detect because of the immediate signs that can be recorded in the milk and udder, making it easier to identify and treat it. On the contrary, SM identification is difficult to detect because of the lack of visible signs in milk or udder and is therefore more persistent on farms [36].

This survey was performed at an average altitude of 3500 m.a.s.l., mostly on small farms and with limited technology. The prevalence of SM at the farm level was 33.7% and that for CM was 5.8%. At the animal level, the prevalence of SM was 35.9% and that of CM was 1.3%. These values are lower than those reported by [37, 38] in Cayambe canton in the Pichincha province in the North of Ecuador where farm SM prevalence was of 54.4% and CM prevalence was 39.47% at the individual level. In the southeast of Ecuador under tropical conditions, SM at the animal level was 60% and 11.6% for CM, following the same pattern [14]; however, they had higher values than this study. Regionally, within some countries of the Americas, the prevalence of SM at the animal level is comparable to that observed in our study, for instance 20.5% in Mexico [6], 33.3% in Peru [20], 46.4% in Brazil [39] and 65.6% in El Salvador [40]. In other regions, the pattern is the same between SM and CM either at the animal level as in India (SM 46% and CM 8%) [1] or at the farm level in Europe where it is 22.7% for SM and 0.6% for CM [33].

The prevalence of SM in this present study is consistently higher than that of CM despite the differences in management, technology and conditions provided by the producer. This is because certain pathogens remain latent in the herd, causing subtle physical changes in the milk that are not easily detectable until CM occurs [41–43]. In contrast, prevalence in different parts of the world means that for every case of CM observed, there are from 15 to 40 cases of SM in the herd [35].

At the animal level, the prevalence of SM was 35.9% and the prevalence of CM was 1.3%. With regard to technological level, in our study, the three technified farms out of 85 had the same ratio of prevalence of SM and CM in relation to farms with limited technological resources (in general farms that had hand milking (82 farms)).

Several studies reported a lower prevalence around different geographic locations in Ecuador: 60% for SM and 11.6% for CM in El Oro [14], South‐West of Ecuador, and for SM 22.22% [44] and 45.5% [37] in Olmedo parish of Cayambe canton in the province of Pichincha‐Ecuador. Conlago & Bonifaz [45] was the only study to report a high prevalence than our study of 66% in the Ayora parish of Cayambe canton in Ecuador. In other regions, the pattern is the same between SM and CM either at the animal level as in India (SM was present in 46% and CM in 8%) [1] or at the farm level as in Europe (22.7% for SM and 0.6% for CM) [33].

A higher number of cases of SM requires more veterinary assistance. Veterinarians assist farmers on general health issues, but these farms do not consider them as part of their staff. Farms rely on veterinarians from public institutions such as MAGAP (Ministerio de Agricultura y Ganadería). However, they help with vaccination or insemination activities, and they are not specifically trained for the milking process or to detect the presence of bovine mastitis. Also, the veterinary assistant might not give pertinent advice that could solve the problem of SM. Improving specific training and capacity building in the prevention of bovine mastitis is an important tool for the farmers and veterinary assistants in the survey area.

The survey also demonstrated that performing the CMT reduces the probability of SM. Although CMT is not a mechanism for preventing bovine mastitis, the fact that farmers are constantly informed about the health of the mammary gland contributes to early action being taken to control the disease. To prevent the disease, this technique has been extensively validated by identifying infected quarters [34, 46, 47]. This method should be generalised as a tool for farmers and veterinary assistants and must be integrated in any future training and capacity building.

This study investigated adequate compliance with mastitis treatment, especially with regard to the withdrawal period for milk from the market due to the presence of antibiotics. Compliance with an appropriate therapeutic regimen is considered a measure to prevent the emergence of new cases of the disease. Therapeutic failures can lead not only to the sick animal not recovering but also to the development of antibiotic resistance in pathogens [48]. In Ecuador, anti‐mastitis products on the market can be divided into two main groups: (1) those used during lactation and (2) those used during the dry period or when there is not yet milk production. In group 1 or lactation, the active ingredients generally used are (A) kanamycin sulphate + benzylpenicillin procaine + prednisolone acetate, (B) spiramycin + neomycin + flumethasone, and (C) amoxicillin + clavulanic acid + prednisolone. In group 2, or drying, the active ingredients are (A) cloxacillin (benzathine), (B) neomycin sulphate + gramicidin + polymyxin B. Lack of training among producers is a contributing factor to the inappropriate use of anti‐mastitis products. This can manifest itself in the administration of insufficient doses, either by the application of a part of the intra‐mammary applicator syringe or by the administration of a single dose instead of those recommended by the manufacturer or the veterinarian.

The only risk factor associated with SM at the farm level was the non‐compliance with medication withdrawal time. The non‐compliance with the time of withdrawal of milk from the markets is mainly due to the lack of knowledge of the producers about the adequate anti‐mastitis treatment schemes. This causes not only the presence of milk with antibiotics in the market but also the inadequate treatment of mastitis cases.

Complying with the waiting time of the medication is part of a therapeutic measure to prevent new cases of SM. In the case of non‐compliance with the treatment time, misusing medication can cause pathogens to develop resistance to antibiotics, making mastitis difficult to treat. Therefore, this risk factor is associated with the incidence of the disease. In addition, we observed that for SM and CM, the presence of CM and SM, respectively, was a risk factor in the univariate analysis. This finding suggests that both SM and CM should be included in all activities of mastitis training and capacity building. This co‐occurrence suggests that both forms of mastitis tend to appear simultaneously or within the same epidemiological context. Surveys routinely find SM far more prevalent than CM, but both occur together. For instance, one study in smallholder herds found 10.5% of cows with CM versus 72% with subclinical infection [49]. Similar patterns were observed in CM with around 3%–21% and SM around 33%–68%. In practice, this means that virtually all herds have some SM (often undetected) even if relatively few cows show clinical signs at any one time. High herd bulk‐tank somatic cell counts (a measure of SCM) are known to correlate with more CM cases. Conversely, cows with recent CM are likely to remain infected and contribute to SM prevalence in subsequent milkings. In short, CM and SM typically go hand‐in‐hand in herds.

According to the Ecuadorian Technical Standards [50], raw milk must meet a series of specific requirements, including organoleptic, physical, and chemical requirements to be considered milk suitable for processing, and numerical ranges are established for each milk component to qualify it. We evidenced the relation between the presence of SM and milk quality characteristics. When the number of SM cases is high, a significantly greater proportion of blood samples show elevated total serum protein. Additionally, milk from affected animals shows decreased density, increased water content and a lower freezing point compared with milk from animals without SM. Indeed, we demonstrated a detrimental effect of SM on the quality of milk produced. The main components such as fat, solid non‐fat and protein can show different degrees of increment or decrement in different types of mastitis when compared to milk from healthy animals [51]. Such alterations in milk quality reduce not only its price but also challenges for the dairy industry in terms of processing efficiency and final product quality [52, 53].

The ROC analysis yielded an AUC of 0.63, indicating a modest ability of the model to discriminate between farms with and without SM. However, the model demonstrated relatively good performance in identifying farms free of SM, with a specificity of 78.6%, suggesting it may be useful for screening purposes or prioritising farms for monitoring. Among the variables included, non‐compliance with medication withdrawal times and performing the CMT had the strongest contributions to the model, along with the low frequency of veterinary assistance, which was a counterintuitive but notable factor. These findings highlight the role of management practices in disease control. Further studies should be stimulated to identify additional risk and protective factors at the farm level to improve the model’s predictive performance and practical utility.

In this survey, at the animal level, the probability of SM presence increased in crossbreeds. Crossbreeding makes new breeds more susceptible to mastitis and was previously found as a risk factor for mastitis in Ethiopia [54]. The finding of our survey should be in favour of a breeding programme to improve production and profitability. However, the environment and the conditions provided to the animals are related and important.

One problem identified on the farms that is worth mentioning is the sale of mastitis medicated milk to the milk plants. In rural areas, producers send milk directly to milk processing plants, while the animals are still in therapy. Producers do this partly because they have limited delivery quotas. If they do not meet these quotas, the companies do not buy their milk back from them. This situation can leave them with no income. Milk with medication is not disposed of, and in some cases, it is destined for self‐consumption. The presence of antimicrobials in milk can impair the production of fermented dairy products by inhibiting starter cultures and, more importantly, expose consumers to drug residues that may trigger allergic reactions or contribute to the development of antimicrobial resistance. Therefore, training based on these results, framed within a One Health perspective, is of vital importance [55].

Finally, reporting all information and results gained during the survey to all survey participants and their family members is a key element to start the process of improving the mastitis situation and antimicrobial resistance.

### 4.1. Limitations of the Study

As this study was conducted in a rural community in the central Highlands of Ecuador, the results may not be representative of other provinces or regions of Ecuador. Furthermore, they cannot be extrapolated to large farms. Similarly, the high prevalence of mastitis among the sampled animals could be related to the health and milking management systems used by producers in the study area rather than to the breed of the animals. Future studies should incorporate different management systems, breeds and levels of technology used in Ecuador’s natural areas (the coast, the highlands, the Amazon region and the islands). The use of new laboratory diagnostic equipment, such as somatic cell counting and milk quality testing, should also be incorporated, as these could be related to mastitis. For example, electrical conductivity could be used as an indicator.

## 5. Conclusions

Bovine mastitis remains a significant health issue in the Ecuadorian highlands, particularly in smallholder dairy systems. This study documented the high prevalence of SM and identified non‐compliance with drug withdrawal periods as a major risk factor, while routine use of the CMT was protective. The apparent association between crossbred cattle and higher mastitis prevalence may reflect the underlying management and resource constraints rather than breed effects per se. SM was also linked to impaired milk quality, including reduced density, added water and lower freezing point.

These findings underscore the need for farmer training, routine CMT screening and strict adherence to drug withdrawal regulations. Strengthening mastitis control and promoting responsible antibiotic use will not only improve herd productivity and milk quality but also contribute to public health and antimicrobial resistance prevention within a One Health framework.

## Disclosure

All authors have read and agreed to the published version of the manuscript.

## Conflicts of Interest

The authors declare no conflicts of interest.

## Author Contributions

Conceptualisation, A.M‐D., M.A.C‐L., J.R‐R. and C.S.; A.M‐D., M.A.C‐L., J.R‐R. and C.S., contributed to the study design; A.M‐D., K.B‐E., M.J.P‐T., C.C‐I., M.A.C‐L., A.R‐B. and J.R‐R. collected field data; A.M‐D. and J.R‐R. verified the underlying data; A.M‐D. conducted the statistical analyses under the supervision of L.R‐G. and C.S.; A.M‐D. drafted the manuscript; C.S., L.R‐G., S.M‐S. and J.R‐R. reviewed and edited the manuscript for clarity.

C.S. and J.R‐R. contributed equally to the work.

## Funding

This survey was funded by the Académie de Recherche et d’enseignement Supérieur (ARES) through the Synergy Project (PRD) entitled ‘Autonomy and training of women as a strategy against cattle mastitis in the centre of highlands of Ecuador’, by the Université de Liège (Belgium) and by the Universidad de las Fuerzas Armadas ESPE (Ecuador).

## Supporting Information

Table S1. Generalised variance inflation factors (GVIFs) for full logistic regression.

This table shows metrics used in full logistic regression model to detect multicollinearity (highly correlated predictors), especially with categorical variables, where GVIF is the generalised variance inflation factor, Df the degree of freedom and GVIF^(1/(2 ∗ Df)) provides a standardised, comparable measure across predictors with different degrees of freedom (Df), allowing standard (VIF) thresholds to be used after squaring the value.

## Supporting information


**Supporting Information** Additional supporting information can be found online in the Supporting Information section.

## Data Availability

The data that support the findings of this study are available from the corresponding author upon reasonable request.
