# Supplementary material for: Prevalence and Farm Management Factors and Milk Quality Changes Related to Bovine Mastitis in Small‐ and Medium‐Sized Farms in the Central Highlands of Ecuador
Source: Vet Med Int. 2025 Dec 29;2025:6213804. doi: 10.1155/vmi/6213804 (PMC12767375; doi:10.1155/vmi/6213804)
Supplement: Supplementary file 1 — Supporting Information Additional supporting information can be found online in the Supporting Information section. [file VMI-2025-6213804-s001.doc]

## **Table S1.** Generalized Variance Inflation Factors (GVIF) for Full Logistic Regression Model

| **Variable** | **GVIF** | **Df** | **GVIF^(1/(2·Df))^** |
| --- | --- | --- | --- |
| Resistant utensils for the milking | 1.6678 | 1 | 1.2915 |
| Sequence of milking protocol | 1.8738 | 1 | 1.3689 |
| Farm Size | 1.6658 | 1 | 1.2907 |
| Enough water for proper milking procedures | 2.2314 | 1 | 1.4938 |
| Frequency of veterinary assistant | 1.9156 | 2 | 1.1765 |
| Separating animals affected with mastitis | 1.8984 | 1 | 1.3778 |
| Performing CMT | 1.4972 | 1 | 1.2236 |
| Training in milking | 2.1765 | 1 | 1.4753 |
| Discard the first milk jet | 1.8194 | 1 | 1.3489 |
| Pre-milking teat washing | 1.3766 | 1 | 1.1733 |
| Teat dipping | 1.5176 | 1 | 1.2319 |
| Non-compliance with medication withdrawal time | 2.1475 | 1 | 1.4654 |
| Mechanised milking | 1.3255 | 1 | 1.1513 |
| Manure presence during milking | 1.4047 | 1 | 1.1852 |
| Clinical mastitis | 1.4027 | 1 | 1.1844 |
